# The role and effectiveness of self-management in a home-based cardiac rehabilitation program: A protocol for systematic review and meta analysis

**DOI:** 10.1097/MD.0000000000020972

**Published:** 2020-07-31

**Authors:** Sisi Zhang, Congying Liang, Jingxian Zhang, Xue Yang, Xiaoping Meng

**Affiliations:** Cardiovascular and Cardiac Rehabilitation Department, First Affiliated Hospital of Changchun Chinese Medicine University, Changchun City, Jilin Province, China.

**Keywords:** anxiety, depression, home-based cardiac rehabilitation, quality of life, self-efficacy, self-management

## Abstract

**Background:**

Home-based cardiac rehabilitation is considered as an alternative strategy of cardiac rehabilitation, aims to enhance patients participation rate. Since it emphasizes patients subjective initiative, patients require a better understanding of their illness and manage their conditions. We perform this systematic review and meta-analysis to identify the role and effectiveness of the self-management program in home-based cardiac rehabilitation.

**Method:**

We conduct the search strategy from an online database: PubMed, web of science, CINAL, EMBASE, OVID/Medline, and google scholar. Studies meet the inclusion criterion and published in the English language in recent 10 years will be screened by 2 independent reviewers. Then they extract data and assess the risk of bias. The disagreement between them will be resolved by the third reviewer. Data analysis is performed by Review Manager (Version 5.3).

**Result:**

This is systematic review and meta-analysis will identify the role of self-management in home-based cardiac rehabilitation program and evaluate its effectiveness.

**Conclusion:**

The findings of this study will be published in a related peer-reviewed journal.

Protocol registration number: INPLASY202050093.

## 1 Introduction

Cardiovascular disease (CVD) has become the leading death in China even worldwide.^[1,2]^ 290 million people suffered from CVD, and 3.72 million died from it in China vs 17.7 million in the global world.^[1,3,4]^ Over the past years, cardiac rehabilitation (CR) has been testified to effectively reduce the mortality, readmissions, secondary events, and improve the health-related quality of life in patients with coronary artery disease.^[5–8]^ At the same time, CR has also been considered a class I recommended by the American Heart Association.^[9]^ Despite the well-known benefits, patient participation remains low, especially within low- and middle-income populations.^[10,11]^ As an alternative strategy of CR, home-based cardiac rehabilitation (HBCR) is a potential approach to help improve rates of participation. Unlike center-based cardiac rehabilitation, HBCR emphasizes the active role of patients and their caregivers. Multiples cost-effective approaches are designed and adapted to deliver this service and improve the quality of HBCR.

Self-management(SM) refers to individual daily work to manage their condition of diseases, symptoms, treatment, lifestyle changes to affect the cognitive-behavioral and emotional responses necessary to maintain a satisfactory quality of life, to reduce the impact of the disease on physical health status.^[12–15]^ It aims to enhance patients ability to managing their illness condition and improving their lifestyle.^[15]^ SM indicate patients active participation, meanwhile emphasize the interactive, collaborative relationship between patients and health professions. The core element of SM is the patients responsibility, which plays an important role in HBCR.^[16]^

In recent years, different kinds of SM programs have been adapted in HBCR, however, some uncertainties exist regarding the role and significance. We carry out this systematic review and meta-analysis aims to

1. identify how many types of SM programs applied to HBCR in the present.
2. assess the role, short, and long-terms effects of SM program compared with usual care program for patients participate in HBCR.

## 2 Method

This systematic review has been registered on the International Platform of Registered Systematic Review and Meta-analysis Protocols with the INPLASY202040057 (https://inplasy.com/). The steps of this systematic review and meta-analysis will be in line with items for systematic review and meta-analysis protocols (PRISMA-P) statement.^[17]^

### 2.1 Search strategy

This review will be carried out using the following database: PubMed, web of science, CINAL, EMBASE, OVID/Medline, and google scholar. All studies about self-management in the HBCR program will be included. The English language will be restricted and all literature will be searched from 2010 to current. We perform the search with the following Medical Subject Heading (MeSH) terms: self-management, self-care home-based cardiac rehabilitation program, home-based cardiac rehabilitation, at home, after the hospital, home care, heart rehabilitation. One example of the search strategy is shown in Table 1, a modification will apply to other databases.

**Table 1:**
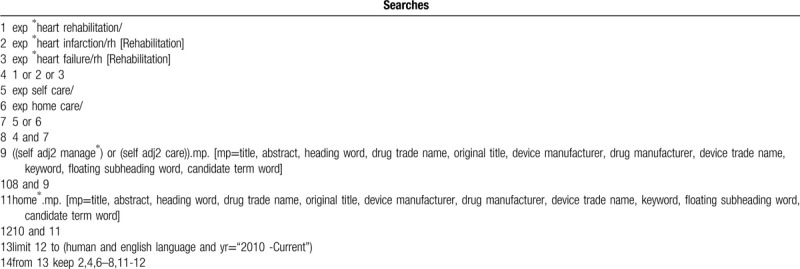
The search strategy of this systematic review.

### 2.2 Study selection

**Figure 1:**
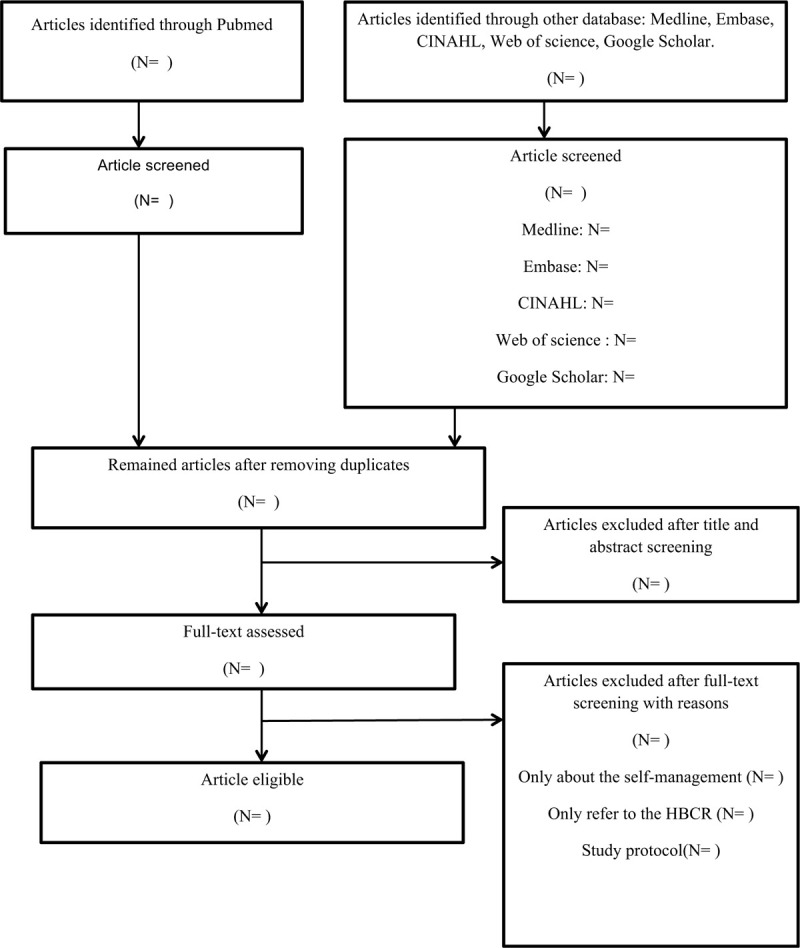
Flow chart of the study selection.

Two reviewers independently and blindly screen the titles and abstracts using inclusion and exclusion criteria, after removing the duplication, the eligible full-texts will be examined. If there is disagreement existing between the 2 reviewers, a senior reviewer will be consulted until a consensus is reached. The process of study selection is shown in a PRISMA flow chart in Figure 1.^[18]^

### 2.3 Selection criteria

#### 2.3.1 Inclusion criteria

The population of the study is adults (age ≥ 18 years). The object of study is about the self-management for patients in HBCR. Published in the English language ranging from January 2010 to December 2019. The studies that meet the criteria are included in the review with no restrictions on study design.

#### 2.3.2 Exclusion criteria

Studies are excluded if the primary focus is not on HBCR or only relate to self-management for chronic diseases. Books, theses, monographs, dissertations, study protocols, abstracts, commentaries, letters, editorial papers, non-human, case reports, unpublished, and non-English researches are also excluded.

### 2.4 Outcome measures

The primary outcome is health-related quality of life (HRQoL), which may be done using the 12-Item Short-Form Health Survey (SF-12), 36-Item Short Form Survey (SF-36), or Minnesota Living with Heart Failure Questionnaire (MLHFQ).^[19,20]^ Other outcomes include self-care behavioral, psychological well-being (Hospital Anxiety and Depression), exercise capacity, self-efficacy, symptoms management, adherence, physician activity, and a 6-minute walking test (6MWT).

### 2.5 Data extraction

Two independent reviews product the data extraction using a standard extraction form, and then in conformity will be resolved by group discussions. The standard form includes

1. Basic characteristics of the included articles: title, first author, year of publication, and country.
2. Study design: randomized control trials (RCTs), observational studies, or cross-sectional studies.
3. Population: target population, sample size, average size.
4. Intervention: type of SM program, design of the program, duration, format, items, and significance.
5. Outcomes: QoL, self-care behavioral, psychological well-being, exercise capacity, self-efficacy, symptoms management, adherence, physician activity, 6MWT, and questionnaires.

### 2.6 Risk of bias assessment

The methodological quality of RCTs included will be assessed by the Cochrane Risk of Bias Assessment Tool, while observational studies will be assessed by the Newcastle-Ottawa scale (NOS) with a score range from 0 (low quality) to 9(high quality).^[22–22]^ Cochrane risk of bias assessment tool includes the following domains: random sequence generation, allocation concealment, blinding of participants and personnel, blinding of outcome assessment, incomplete outcome data, selective reporting, and other possible biases, each of them is classified into the high, unclear or low risk of bias.

### 2.7 Data synthesis and statistical analysis

Data synthesis will be conducted with Review Manager (Version 5.3). Continuous data perform with standardized mean differences (SMD) and 95% confidence intervals (CI).^[23]^ X^2^ test and *I*^2^ statistics are used to assess the heterogeneity, with *I*^2^ statistics ≥50% and *P* < .10 are considered as substantial heterogeneity.^[24]^ We adapt the fixed effects model if the heterogeneity is low, otherwise using the random-effects model.

### 2.8 Subgroup analysis

Subgroup analysis will be performed if adequate data are available in terms of different kinds of SM program and duration time.

### 2.9 Evidence evaluation

The quality of evidence is assessed by the Grading of Recommendations, Assessment, Development, and Evaluation (GRADE) system, with a classification of “high”, “moderate”, “low” or “very low” quality.^[25]^

## 3 Discussion

To our knowledge, this is the first systematic review, and meta-analysis identifies SM programs in HBCR and evaluates its effects. These outcomes will provide more evidence for future studies, thus help improve the quality of management in HBCR. However, there are also some limitations existing. Firstly, the literature search only limits in recent ten years with the English language, which will exclude some other studies. Secondly, heterogeneity maybe exists in different SM programs.

### 3.1 Uncited reference

^[21]^.

## Author contributions

Ss Z conceives and designs this protocol and register on PROSPER. Ss Z, Jx Z and Cy L conduct the search strategy and data extraction. Ss Z and XY are responsible for writing this protocol, Xp M revise the protocol during the whole stage.
